# In Utero Diagnosis of Agenesis of the Ductus Arteriosus in a Twin Pregnancy: An Unusual Case Presentation

**DOI:** 10.5402/2011/258431

**Published:** 2010-12-01

**Authors:** Kecia Gaither, Andrea Ardite, Sarita Dhuper

**Affiliations:** ^1^Department of Obstetrics and Gynecology, Brookdale University Hospital and Medical Center, 1 Brookdale Plaza, 4 Katz, 4C-Rm 436, Brooklyn, NY 11212, USA; ^2^Department of Obstetrics and Gynecology, Division of Maternal Fetal Medicine, Brookdale University Hospital and Medical Center, Brooklyn, NY 11212, USA

## Abstract

Agenesis of the ductus arteriosus is a rare congenital cardiac anomaly which should be considered within the differntial prenatal diagnosis of hydrops fetalis.

## 1. Introduction

Agenesis or premature closure of the ductus arteriosus is an uncommon anomaly. The defect, when present, is usually associated with Tetrology of Fallot (TOF), absent pulmonary valve, truncus arteriosus, or maternal use of prostaglandin synthetase inhibitors for the arrest of premature labor.

Isolated premature closure of the ductus with or without congenital heart disease can result in right sided heart failure and fetal hypoxemia resulting in hydrops fetalis. We present a case involving the prenatal diagnosis of agenesis of the ductus arteriosus and Tetrology of Fallot complicating a twin pregnancy.

## 2. Case Report

A 34-year-old African American woman Gravida-4, Para-3, presented for prenatal care at 15 weeks of gestation by her last menstrual period. She had no significant pastobstetric, medical, or family history. Comprehensive sonographic evaluation at 18 weeks gestation revealed a diamniotic dichorionic twin gestation, cleft lip in Twin B, and large echogenic intracardiac foci in the left ventricles of both twins. A fetal echocardiographic study revealed the following. Twin A was noted to have an echogenic focus in the left ventricle with evidence of ventricular septal defect, overriding aorta, and a small dysplastic pulmonary valve with pulmonary regurgitation. Twin B was also noted to have an echogenic focus in the left ventricle with evidence of ventricular septal defect, overriding aorta, and a small pulmonary valve characteristic of TOF. The patient underwent genetic consultation, but declined amniocentesis for kayotypic analysis. Subsequent echocardiographic evaluation performed at 27 weeks gestation revealed the following. Twin B was noted to have decreased right ventricular function, bradycardia with absent end diastolic flow in the umbilical artery and hydrops. Twin A was also noted to have frequent episodes of bradycardia. Due to the findings of fetal hydrops, and nonreassuring antenatal testing, a cesarean section was performed after administration of steroid therapy.

**Figure 1:**
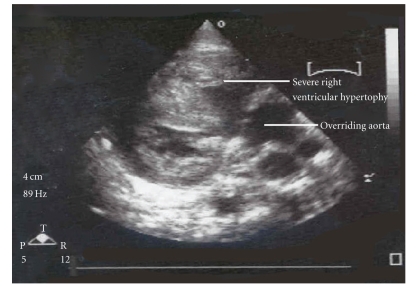
Echocardiogram of Twin B showing overriding aorta and right ventricular hypertrophy.

**Figure 2:**
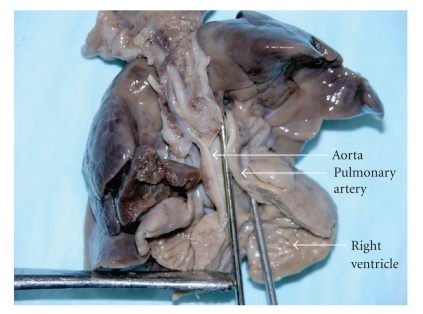
Autopsy of Twin B heart and lungs showing small sized pulmonary artery and right ventricular hypertrophy.

At birth, Twin B weighed 820 grams and was noted to have a cleft lip and penile hypospadias; Apgars at one and five minutes were of 4 and 6, respectively. The infant was subsequently electively intubated and ventilated. Initial arterial blood gas revealed mild metabolic acidosis. Cardiac evaluation revealed the patient to be in sinus rhythm and echocardiography confirmed the diagnosis of Tetralogy of Fallot with mild right ventricular outflow tract obstruction; no ductal shunt was appreciated (Figure 1). On day two of life, the neonate was noted to have decreasing oxygen saturation associated with hypotension and metabolic acidosis. Echocardiogram revealed increasing infundibular stenosis and right ventricular outflow tract gradient above 40 mm Hg. Ductal flow was still not demonstrated which was unusual given the gestational age and development of hypoxemia. Dopamine at 10 microgram/kg/min and Prostaglandin E-1 (PGE-1) at 0.05 microgram/kg/min was initiated with minimal clinical improvement. Cardiothoracic consultation from an adjacent tertiary care center was requested, however given the patient's weight and unstable condition surgical intervention was not considered an option. There was progressive deterioration in the form of oliguric renal failure with anasarca and severe metabolic acidosis and the infant expired on day 7 of life. Karyotyping revealed 46 XY genotype with no evidence of micro- or macrodeletions of long arm of chromosome-22. Autopsy confirmed the ante-mortem cardiac findings of TOF and the pulmonary artery was markedly diminished in size. The ductus arteriosus could not be demonstrated (Figure 2).

**Figure 3:**
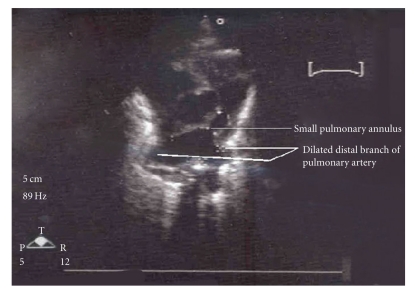
Echocardiogram of Twin A showing small pulmonary annulus and dilated distal branch of pulmonary artery.

Twin A at birth weighed 790 grams, had one and five minute Apgars of 4 and 6, respectively and was also electively intubated and ventilated. Arterial blood gas did not reveal any metabolic acidosis. Echocardiography confirmed Tetrology of Fallot and pulmonary regurgitation. The main pulmonary artery annulus was small and measured 2.5 mm and the distal branch of pulmonary arteries measured 4.5 mm (Figure 3). No flow was appreciable across the ductus arteriosus. Initially oxygen saturations were within low normal range, however after four days of life, the neonate began to have decreasing values. Echocardiogram revealed increasing subinfundibular and valvar stenosis with gradients increasing to 60–80 mm hg over time. PGE-1 drip at 0.1 mcg/kg/min was initiated with no significant improvement in the saturations and no ductal flow was demonstrated on the echocardiogram. As with Twin B, cardiothoracic surgical intervention was sought but declined given the high risk. The patient's clinic course progressively worsened with progressive metabolic acidosis, cardiac decompensation, and sepsis. The infant expired on day 30 of life. Normal 46 XY karyotype was noted. The parents declined autopsy.

## 3. Discussion

Absence of the ductus arteriosis was first described in 1671 by Stensen in a fetus with TOF [1, 2]. Most such cases of TOF with absent ductus have the associated absent pulmonary valve syndrome and aneurysmally dilated pulmonary arteries. [3, 4]. The usual presentation with this condition is increasing respiratory distress due to compression of the bronchi. [3]. Another form of presentation is heart failure or hydrops diagnosed in utero [5, 6].

Both our patients had TOF variants but presented with progressive cyanosis not responding to PGE 1 due to absent ductus arteriosus. Twin A had TOF with a dysplastic pulmonary valve with moderate stenosis and regurgitation but over time developed increasing infundibular stenosis, cyanosis and right ventricular failure rather than progressive respiratory distress as is typically described in the TOF/absent pulmonary valve syndrome.

Twin B had typical features of TOF but presented with right ventricular dysfunction and early hydrops requiring urgent delivery. Absent ductus arteriosus was suspected only after birth, when there was no response to prostaglandin. Both patients became progressively cyanotic over time and failed to improve with increasing doses of PGE-1. 

This case brings to the literature the association of TOF and absent or premature closure of the ductus not associated with usual pulmonary regurgitation and aneurysmal pulmonary arteries. This association should be considered and looked for specifically during in utero studies as premature delivery may not be a viable option as was in our case. Although we were not able to do so in our case, it is possible to make the diagnosis of ductal closure in utero by Doppler flow studies as described by Huhta et al. [7]. Moreover, if congenital heart disease is associated with myocardial dysfunction especially of the right ventricle and hydrops, absent ductus arteriosus should be considered, as it is not usual for congenital heart disease to be associated with cardiac dysfunction unless there is an associated arrhythmia, heart block, or myocarditis. The case emphasizes the need to consider absent or premature ductal closure in a fetus with hydrops of unknown etiology and especially associated with any variant of TOF.
